# Graphene and its Derivatives for Bone Tissue Engineering: *In Vitro* and *In Vivo* Evaluation of Graphene-Based Scaffolds, Membranes and Coatings

**DOI:** 10.3389/fbioe.2021.734688

**Published:** 2021-09-29

**Authors:** Junyao Cheng, Jianheng Liu, Bing Wu, Zhongyang Liu, Ming Li, Xing Wang, Peifu Tang, Zheng Wang

**Affiliations:** ^1^ Department of Orthopaedics, Chinese PLA General Hospital, Beijing, China; ^2^ Chinese PLA Medical School, Beijing, China; ^3^ Beijing National Laboratory for Molecular Sciences, Institute of Chemistry, Chinese Academy of Sciences, Beijing, China; ^4^ University of Chinese Academy of Sciences, Beijing, China

**Keywords:** graphene, bone tissue engineering, scaffolds, membranes, coatings

## Abstract

Bone regeneration or replacement has been proved to be one of the most effective methods available for the treatment of bone defects caused by different musculoskeletal disorders. However, the great contradiction between the large demand for clinical therapies and the insufficiency and deficiency of natural bone grafts has led to an urgent need for the development of synthetic bone graft substitutes. Bone tissue engineering has shown great potential in the construction of desired bone grafts, despite the many challenges that remain to be faced before safe and reliable clinical applications can be achieved. Graphene, with outstanding physical, chemical and biological properties, is considered a highly promising material for ideal bone regeneration and has attracted broad attention. In this review, we provide an introduction to the properties of graphene and its derivatives. In addition, based on the analysis of bone regeneration processes, interesting findings of graphene-based materials in bone regenerative medicine are analyzed, with special emphasis on their applications as scaffolds, membranes, and coatings in bone tissue engineering. Finally, the advantages, challenges, and future prospects of their application in bone regenerative medicine are discussed.

## Introduction

For the past decades, the incidences of bone defects caused by different musculoskeletal conditions (e.g., congenital, degenerative, post-traumatic, neoplastic, metabolic and infectious) are continuously increasing (Katagiri et al., 2017). Bone regeneration or substitution has been proved to be valid approaches for the current therapy (Bhattacharjee et al., 2017; Zhang et al., 2019a). In this context, bone transplantation has become the second frequent tissue transplantation after blood transfusion, with over two million cases worldwide per year (Liu et al., 2017). With the advent of an aging society, the clinical requirement for effective bone regeneration therapy will continue to increase. Despite autogenous bone transplantation has undoubtedly become the gold standard for bone regeneration, the applications are still limited because of the insufficient supply, loss of function as well as secondary defects at the bone donor site (Shukla et al., 2017; Benlidayi et al., 2018; Zhang et al., 2019b). Allogenous bone transplantation is an alternative approach, but this therapeutic method has to face risks such as disease transmission, infection, and immunogenicity (Calori et al., 2011; Campana et al., 2014). Although some existing synthetic biomaterials have achieved favorable clinical efficacy, there is still a lack of outstanding biomaterials that can provide excellent load-bearing, complete biodegradability, osteogenesis and osteoconductivity simultaneously (Fayyazbakhsh et al., 2017; Li et al., 2018a; Ju et al., 2021). As a result, there has been an urgent need for the development and application of synthetic bone graft substitutes.

Tissue engineering has been considered as a viable solution to the aforementioned challenges, which has led to significant advances in cell and organ transplantation over the past decades, as well as greatly stimulating innovation in new materials, application models, preparation techniques, and performance evaluation (Rose and Oreffo, 2002; O'Keefe and Mao, 2011). Tissue engineered bone grafts have great potential to alleviate the need arising from the lack of suitable autograft and allograft materials for bone repair (Yadav et al., 2021). Up to now, a great deal of effort has been made in the design, fabrication, characterization, and application of emerging materials, such as scaffolds, coatings, membranes. (Zwingenberger et al., 2012; Eivazzadeh-Keihan et al., 2019; Du et al., 2021). Among these promising solutions, scaffolds play a central role and exhibit several unique advantages in bone tissue engineering (Shadjou and Hasanzadeh, 2016). Scaffolds can not only provide mechanical support for local load-bearing, but also offer structural support for specific cells, and thus guide new tissue growth and promote bone regeneration (Sill and von Recum, 2008). Coatings for bone repair implants are perceived as another promising approach in bone tissue engineering. Coated materials can enhance the mechanical properties of the implants and improve the interfacial reaction physiologically (Pereira et al., 2020). Membranes for bone tissue engineering can provide an independent space for bone regeneration and act as a barrier against soft tissue ingrowth (Du et al., 2020).

The success of tissue engineering heavily depends on the performance of the materials. For the desired bone regeneration materials, excellent biocompatibility, controlled biodegradability, appropriate mechanical strength, and suitable porosity to support cell differentiation, growth, and proliferation all should be emphasized (Kim et al., 2013; Freeman et al., 2021; Hajiali et al., 2021). Research on graphene-based nanomaterials has boomed in biomaterial applications over the past few years. With outstanding physical, chemical and biological properties, graphene is considered to be revolutionary material and shows great potential for applications in tissue regeneration, drug delivery and other biomedical areas (Hu et al., 2020; Fu et al., 2021; Unal et al., 2021). Therefore, the purpose of this review is to highlight the scientific progress over the years and further summarize the physical and chemical properties, family members and applications in bone tissue engineering of this graphene-based nanomaterial.

## Advantages of Graphene in Bone Tissue Engineering

Graphene, a single-atom thick and two-dimensional sheet of sp^2^-hybridized carbon atoms, was isolated from highly oriented pyrolytic graphite by two British physicists (Feng and Liu, 2011). This revolutionary discovery quickly attracted great attention in the fields of materials science, chemistry, physics and biotechnology, and the 2010 Nobel Prize in Physics attests to its extraordinary significance (Dresselhaus and Araujo, 2010). Since its discovery in 2004, graphene has received increasing interest for its remarkable properties, including high fracture strength, outstanding Young’s modulus, excellent thermal and electrical conductivity, large specific surface area, atomic structure stability and biocompatibility (Kumar et al., 2016; Sayyar et al., 2017; Zhao et al., 2017; Nezakati et al., 2018). There have been great expectations for the application of this “future material”.

Graphene is known as one of the strongest materials in existence (Lee et al., 2008). With exceptional mechanical properties, graphene stands out as the most promising candidate to be a major filling agent for bone repair composite. Nevertheless, it should be emphasized that the mechanical reinforcement effect from graphene is closely related to its distribution in the composite (Young et al., 2012). Homogeneous distribution leads to effective mechanical property enhancement, but the cohesion between graphene molecules can hinder the distribution and therefore needs to be overcome (Gao et al., 2018). Electrical conductivity can confer better osteogenic activity to bone repair materials (Huang et al., 2019). Owing to its unique molecular structure, graphene can be used to formulate three-dimensional composites with good electrical conductivity. Moreover, the large specific surface area of graphene can greatly improve cell adhesion, which likewise benefits the osteogenic activity (Gao et al., 2017). Large specific surface area also facilitates the further functionalization of graphene, thus being able to impart better chemical activity and improve its hydrophilicity and dispersibility (Trusek et al., 2020). The molecular size, content and uniformity of graphene will significantly affect the mechanical and electrical properties of the bone repair composite, and it is therefore important to determine the appropriate graphene content and ratio.

Biocompatibility is a prerequisite for the *in vivo* application of bone repair materials (Mehrali et al., 2014; Qi et al., 2020; Jyoti et al., 2021). A number of studies have demonstrated the biocompatibility of graphene through *in vitro* cell co-culture and *in vivo* metabolic analysis, however, this observation is accompanied by qualifying conditions (Alaghmandfard et al., 2021). The physical and chemical properties have been proved to greatly affect the interaction of graphene with living cells, and that the dose and concentration of graphene in the matrix are also related to its cytotoxicity (Cao et al., 2021; Pulingam et al., 2021). The resulting cytotoxic effects occur mainly at the cellular and molecular levels and may be attributed to increased oxidative stress (Sasidharan et al., 2016). Therefore, as with other nanomaterials, smaller particle sizes and higher concentrations are more likely to induce cytotoxic effects, while concentrations below 5–10 μg/ml are relatively safe (Chang et al., 2011). The long-term safety of biomaterials for *in vivo* applications also depends on their biodegradability. The degradation products from graphene have not been shown to cause substantial cell damage, however, the *in vivo* retention period may be associated with several pathological changes (Mukherjee et al., 2018; Daneshmandi et al., 2021). The enzymatic environment as well as specific chemical modifications are available to regulate the rate of degradation and thus reduce the potential of graphene-induced cytotoxicity (Palmieri et al., 2019; Ma et al., 2020; Peng et al., 2020).

## Derivatives of Graphene

The growing demand for solving clinical challenges is forcing the studies on tissue engineering to progress (Wang et al., 2021). As one of the most promising emerging materials, graphene has been widely explored in the fields of regenerative medicine (Kolanthai et al., 2018; Liu et al., 2019; Cojocaru et al., 2021). Nevertheless, there are still much room for improvement to overcome existing challenges for its application in bone regeneration (Cernat et al., 2020; Nimbalkar and Kim, 2020; Bruschi et al., 2021). In spite of remarkable properties, it is difficult for pristine graphene to form three-dimensional scaffolds on its own. In general, graphene needs to be compounded with other materials for bone repair (Raslan et al., 2020), but the strong van der Waals forces between particles lead to the poor dispersibility in aqueous media and physiological fluids (Li et al., 2017a). Functionalization of pristine graphene is an effective method to improve the solubility and dispersibility, so that a growing number of studies have been focusing on its derivatives for regenerative applications in recent years (Driscoll et al., 2021).

Graphene oxide (GO) is the oxidized form of graphene, which has been the most widely used graphene family member in biomedical applications today (Reina et al., 2017; Han et al., 2018a; Martín et al., 2019). After oxidation, GO retains its laminar structure. There are various oxygen functional groups distributed on the carbon atom sheet, mainly including hydroxyl and epoxy groups on the basal plane, carboxyl and carbonyl groups attached to the edge (Park et al., 2009). The introduction of functional groups not only offer the hydrophilicity and dispersibility (Chen et al., 2012), but also provide more opportunities to manipulate and customize the properties of GO (Kim et al., 2010). On the other hand, the presence of these functional groups produces high defect density in the perfect planar structure of graphene, which leads to the reduction of its mechanical, electrical, and thermal properties (Zhao et al., 2021). Therefore, GO is considered as an attractive and cost-effective alternative for graphene due to its accessibility, hydrophilicity, dispersibility, chemical tunability and processability.

Reduced graphene oxide (rGO) is obtained through chemical or physical methods to eliminate the oxygen functional groups (Dash et al., 2021). Reduction of GO is done to restore physical properties to some extent, but this also leads to a decrease in hydrophilicity and dispersibility, as well as a weakening of the chemical tunability (Bagri et al., 2010). rGO can be used as an alternative for large-scale production of graphene-based materials. Based on number of layers in the sheet, graphene can be classified into single layer graphene, few-layers graphene and multi-layer graphene (Daneshmandi et al., 2021). Ultimately, the application of graphene and its various functionalized derivatives can be selected according to specific clinical needs in bone regenerative medicine (Zhao et al., 2017). Currently, graphene and its derivatives (GDs) have been used to prepare various scaffolds, coatings, membrane materials and injectable hydrogels for bone tissue engineering by compounding with various matrices such as metals, polymers and inorganic substances (Park et al., 2016; Li et al., 2018b; Saravanan et al., 2018).

## Applications of GDs in Bone Tissue Engineering

It is necessary to have a thorough understanding of the basic structure before discussing bone tissue engineering. In addition to bone cells, bone tissue contains a large number of matrices, mainly including collagen, non-collagenous proteins and calcium phosphate deposits (Katz and Meunier, 1987). Macroscopically, bone tissue can be divided into dense cortical bone and porous cancellous bone or trabecular bone. In general, cortical bone acts as a shell to encase cancellous bone. Tissue regions with higher mechanical stress contain a higher percentage of cortical bone (Rho et al., 1998). It can be assumed from the composition contained in bone tissue, the mineral component provides stiffness while the collagen assemblies provide viscoelasticity and toughness (Kadler et al., 1996; Iyo et al., 2004; Shah et al., 2019). Microscopically, pristine fibers, formed from collagen and minerals, can aggregate and assemble into nanoscale fibers. Subsequently, the nanoscale fibers gathering as lamellar structures that are arranged in cylinders parallel to the long axis in cortical bone and irregularly woven arrays in cancellous bone (Rho et al., 1998). Bone tissue is also a highly dynamic system. Continuous remodeling occurs through osteolysis by osteoclasts and osteogenesis by osteoblasts (Hadjidakis and Androulakis, 2006). The process of bone remodeling can be induced by mechanical stress, with areas of higher mechanical stress producing stronger tissue and higher overturn rates (Herring, 1968).

### GDs-Based Scaffolds

Synthetic scaffolds have become attractive alternative to natural graft materials due to their accessibility, affordability, adjustability and stability (Damien and Parsons, 1991). In addition to immediately restoring the mechanical integrity at the bone defect site, the ideal biodegradable scaffolds should also provide spaces to guide new bone tissue growth and reconstruction (Kim and Mooney, 1998). The selection of suitable materials for the fabrication of high-quality three-dimensional porous scaffolds is quite an important issue in bone tissue engineering. On account of the ability to significantly improve mechanical and biological properties in the field of synthetic scaffolds, significant research has focused on GDs in recent years.

**FIGURE 1:**
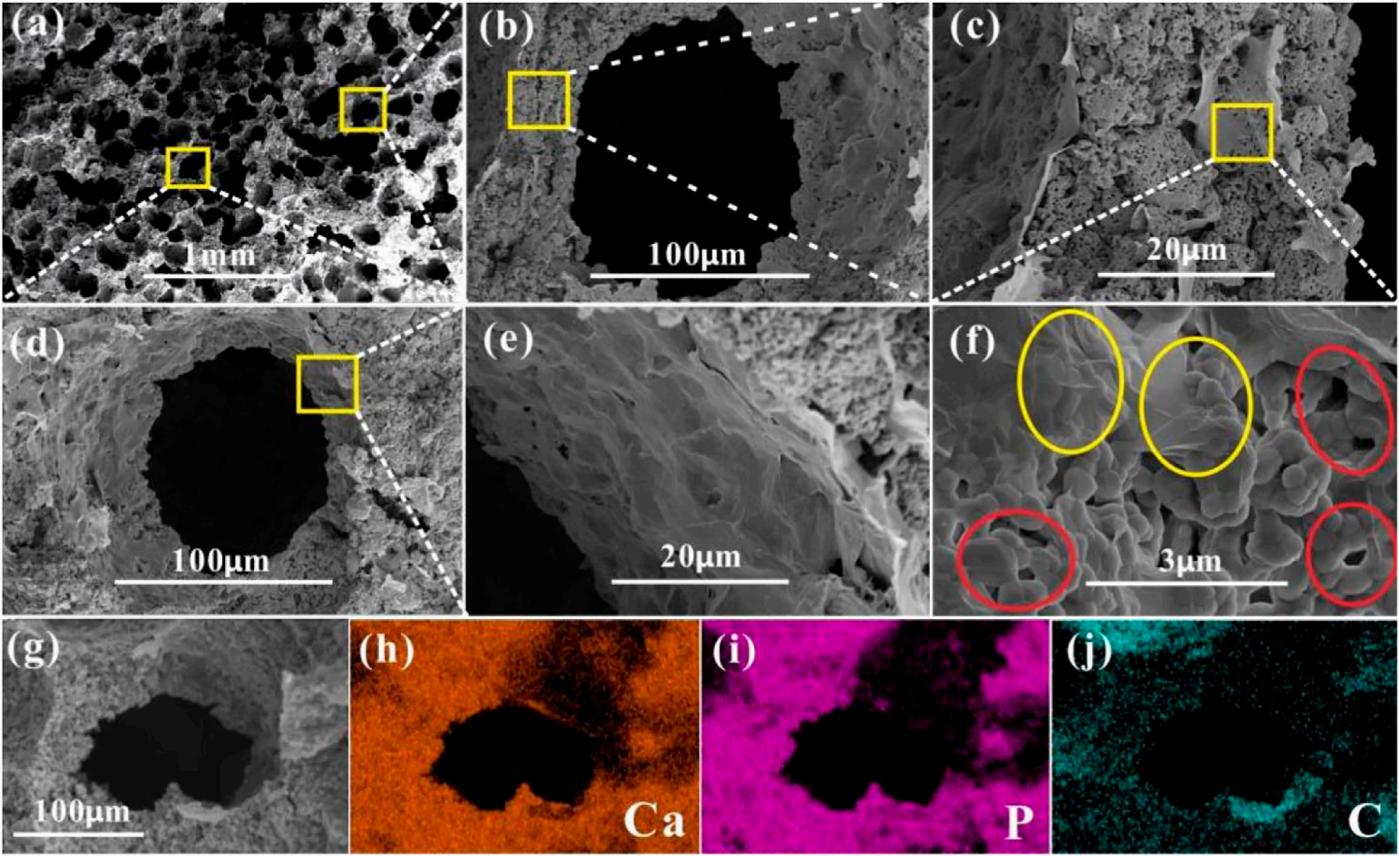
SEM image of **(A)** the porous structure in HA/rGO-6/0.3 composite scaffold, and **(B) (D)** the pore structure, **(C)** cross-sectional structure of hole wall, **(E)** pore wall structure and **(F)** enlarged view of the cross-section of hole wall. **(G)** Pore structure of HA/rGO-6/0.3 and EDS **(H)** Ca, **(I)** P **(J)** C mapping images of Figure 2G. Reproduced from Zhou et al. (2019) with permission from Copyright 2019 American Chemical Society.

Hydroxyapatite (HAP), due to its similarity to the inorganic composition of bone tissue and good biocompatibility, was the most popular bone replacement material. However, its low fracture toughness, low wear resistance, brittleness, and poor osteoinductive ability greatly limit the application, which needs to be solved urgently (Shin et al., 2015). Zhou et al. (2019) successfully prepared a porous scaffold with hierarchical pore structure and good biomechanical strength using a soft template method. With HAP as substrate of the scaffold, rGO was introduced to improve mechanical properties and promote proliferation and spontaneous osteogenic differentiation of bone marrow mesenchymal stem cells (BMSC). More importantly, it allowed the rate of scaffold degradation to closely match the rate of new bone growth. The hierarchical porous HAP/rGO composite scaffolds was proved to accelerate bone growth in the scaffold, providing a potential clinical candidate for regeneration of critical bone defects (Figure 1). To avoid HAP agglomeration affecting the overall bioactivity and stability, Zhao et al. (2019) introduced nano-hydroxyapatite (nHAP) into the chitosan (CS)/GO covalently-bound network matrix. The covalent bonding between CS and GO provided the underlying stability of the scaffold. The nanoscale network substrate could promote uniform dispersion of nHAP, reinforcing the interactions between organic and inorganic materials, which further increased the overall bioactivity and stability. The lamellar structure of GO created a certain spacing between the composite units, thus enhancing the hydrophilicity of the scaffold. The abundant functional groups on the surface of GO and CS promoted the recruitment, proliferation and differentiation of endogenous stem cells. *In vitro* experiments demonstrated that these GO/CS/nHAP scaffolds achieved excellent endogenous bone tissue regeneration, and the new bone formed an almost complete structure with the surrounding natural bone.

**FIGURE 2:**
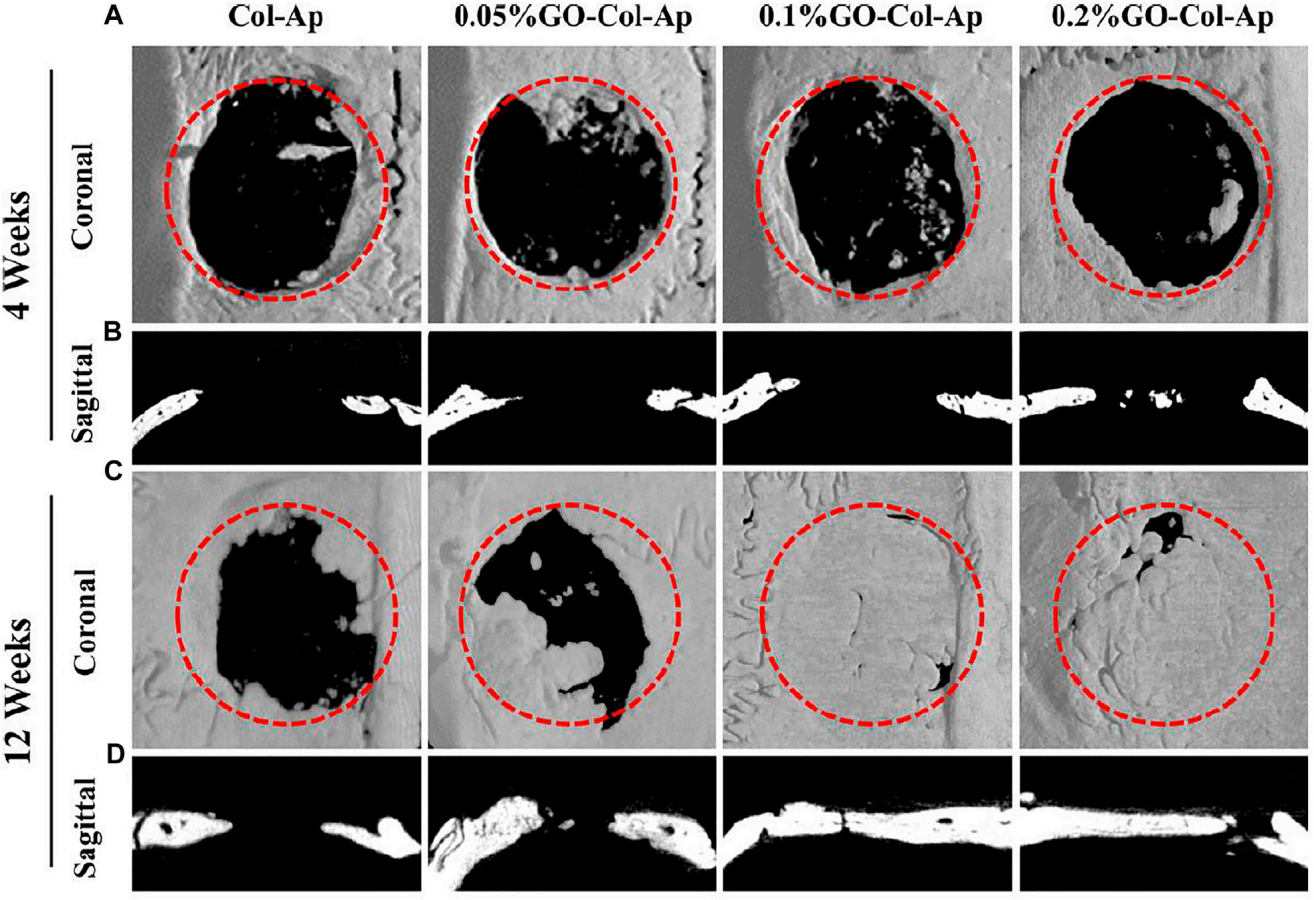
*In vivo* evaluation of the scaffolds using the critical-sized defect **(A, C)** Three-dimensional reconstruction and **(B, D)** coronal section analysis of the defect areas at 4 and 12 weeks **(A, B)** New bone was formed in the four groups at 4 weeks, and **(C, D)** almost complete healing of the bone defects was observed in 0.1 and 0.2% GO−Col−Ap groups at 12 weeks. Dotted red circle: defect area. Reproduced from Zhou et al. (2018) with permission from Copyright 2018 American Chemical Society.

Collagen is an organic component of the bone tissue matrix that is widely used in bone tissue engineering (Ahn et al., 2021). Biocompatibility, biodegradability, bioactivity, and low immunogenicity are the advantages of collagen-based scaffolds. However, due to insufficient mechanical strength, they usually need to be used in conjunction with other materials for bone reconstruction, wherein GDs are one of the ideal choices (Sarker et al., 2015). Taking advantage of the biocompatibility of collagen and GO, Liu et al. (2018) constructed a novel scaffold that mimic the extracellular matrix environment of BMSC. An osteoinductive extracellular matrix (OiECM) was obtained by incubation of osteo-differentiated BMSC for 21 days. Then the OiECM was completely wrapped with GO-collagen (Col) hybrids to construct the OiECM-GO-Col scaffold. The excellent bone repair effect of the new scaffold was demonstrated using a 5 mm rat cranial defect model. In addition, Zhou et al. intended to form bone-like apatite (Ap) on natural polymers through biomimetic mineralization using simulated body fluid (SBF), thus enhancing the osteoconductivity and biocompatibility (Zhou et al., 2018). To improve the coating efficiency of the bone-like apatite layers, GO, which is rich in functional groups, was utilized to provide more active sites for biomimetic mineralization. Different concentrations (0, 0.05, 0.1, and 0.2% w/v) of GO were introduced into the collagen scaffold, and the fabricated scaffolds were then immersed into SBF for 1, 7, and 14 days. Through a series of experiments, it was observed that the 0.1% GO-Col-Ap group formed more bone-like apatite and showed significantly higher rat BMSCs adhesion and proliferation *in vitro* and higher bone formation *in vivo* (Figure 2).

**FIGURE 3:**
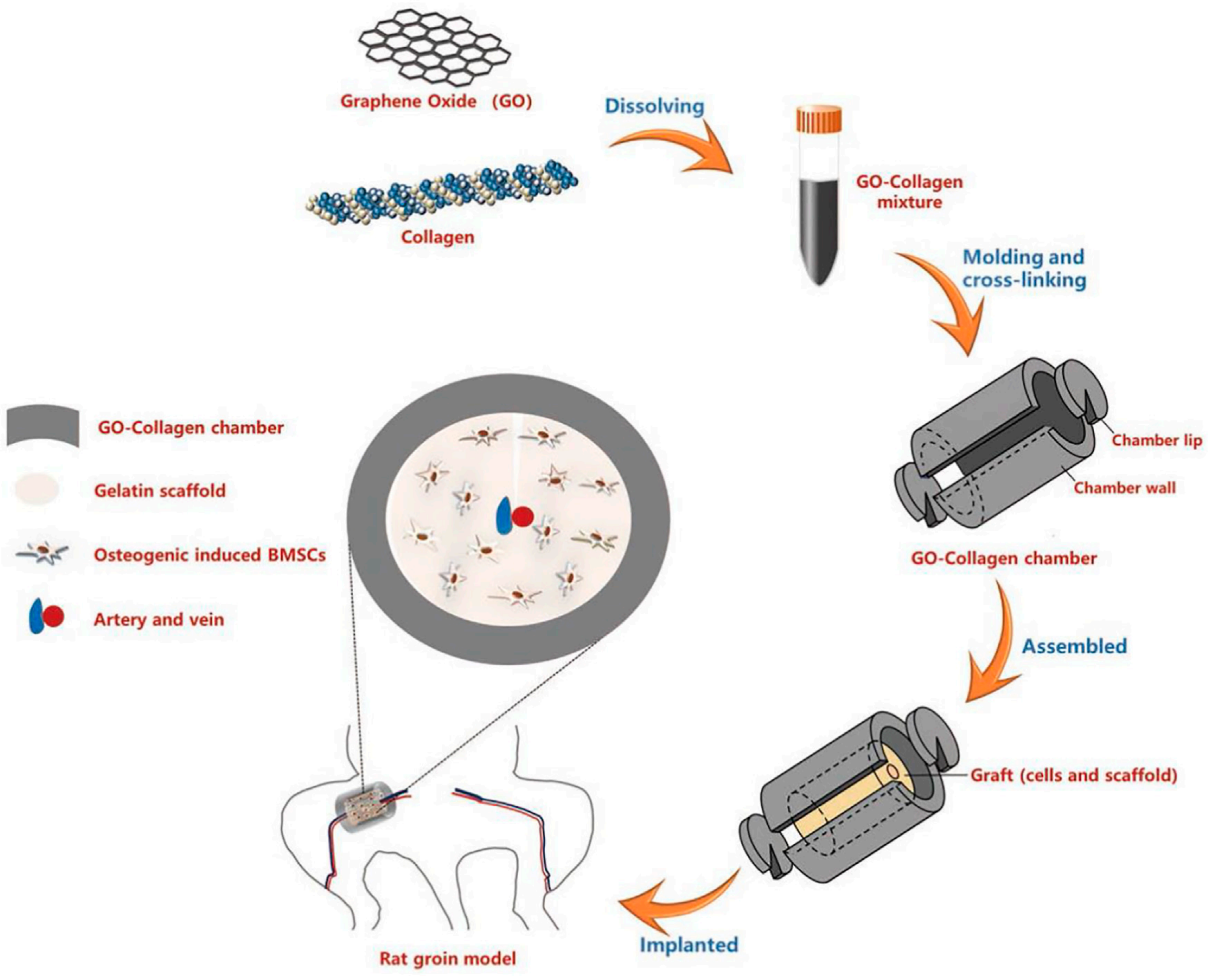
Schematic illustration of the preparation and *in vivo* application of the GO-collagen tissue engineering chamber in a rat groin model. GO and collagen were dissolved, blended and injected into molds to obtain GO-collagen scaffolds with disc shape and hollow cylindrical shape. After the cross-linking process, GO-collagen scaffolds were fabricated to make a tissue engineering chamber. Then, the BMSCs-gelatin grafts were encased in the GO-collagen chamber and implanted into the rat groin area, with vessels traversing through the graft. Reproduced from Fang et al. (2020) with permission from Copyright 2020 Ivyspring International Publisher.

A microenvironment with adequate blood supply is equally crucial for bone tissue engineering (Yap et al., 2018). Some implant failures were caused by the lack of nutrients secondary to insufficient vascularity (Auger et al., 2013). Wang et al. (2019) designed a scaffold comprising of mesoporous bioactive glass (MBG) and GO to investigate its ability to promote local angiogenesis and bone healing. In a rat cranial defect model, the MBG-GO scaffold demonstrated its ability to promote inward vascular growth. The osteogenic-angiogenic properties made this novel material as an attractive candidate for bone repair. For example, Zhao et al. (2018) developed a novel thermosensitive injectable scaffold material via combination of GO with a citrate-based hydrogel called PPCNg. BMP9-encapsulated GO-PPCNg scaffold greatly enhanced the expression of osteogenic regulators, bone markers and vascular endothelial growth factor (VEGF). Moreover, the formation of well-mineralized and highly vascularized trabecular bone was observed *in vivo*. Tissue engineering chamber is an *in vivo* transplantation device that cannot only provide the mechanical support for transplanted tissue or cells but also endow a relatively isolated and vascularized environment (Rouwkema et al., 2008). Fang et al. (2020) established a vascularized GO-collagen chamber by embedding blood vessels into the internal BMSCs-gelatin grafts (Figure 3). After placement in the inguinal region of rats for 1 month, GO-collagen chambers were shown to significantly improve the angiogenic process and promote the survival and osteogenic differentiation of BMSCs.

### GDs-Based Membranes or Films

Artificial barrier membranes can seal bone defects and promote bone regeneration during a regenerative period of up to several weeks, and have likewise attracted a lot of attention in bone tissue engineering (Retzepi and Donos, 2010). Previous studies have reported the use of titanium, polymers and some bioactive materials for fabrication of bone repair membranes (Molly et al., 2006; Gentile et al., 2011; Tejeda-Montes et al., 2014). Its properties are yet to be improved, especially mechanical properties matching the bone tissue, osteogenic activity, controlled degradability and selective permeation of nutrients (Lee et al., 2009; Pinheiro et al., 2009; Choi et al., 2010). During regenerative period, the bone repair membrane not only acts as a framework allowing new bone formation, but also provides a sealed space to prevent rapid ingrowth of connective tissue (Zhang et al., 2021).

**FIGURE 4:**
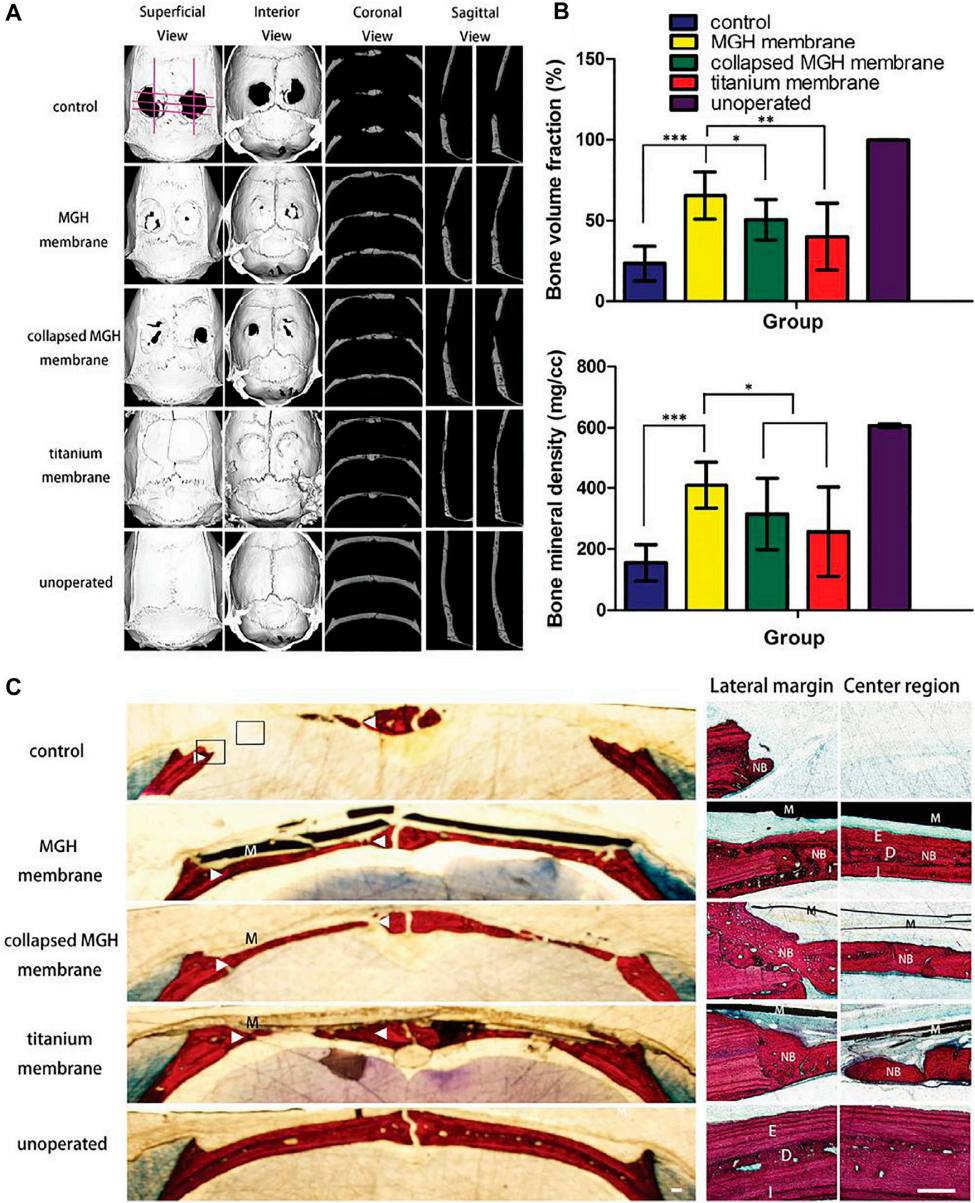
The bone regeneration 8 weeks after surgery. **(A)** Micromorphometric analysis of treated calvarial defects including superficial, interior, coronal, and sagittal section views of micro-CT images taken at the eighth week after surgery. **(B)** Micromorphometric bone parameters including bone volume fraction and bone mineral density analyzed after 8 weeks of surgery. Note that both the bone volume fraction and mineral density of the MGH membranes group are higher than the rest of the groups analyzed. **(C)** Van Gieson’s staining of calvarial undecalcified sections after 8 weeks of implantation. Low-magnification histological images **(left)** showed osteogenesis of the testing groups with/without barrier membranes (M). High magnification histology **(right)** showed boxed areas in the left images, both the lateral margin and center region of defects. In the MGH membrane group, the newly formed bone (NB) exhibited a mature lamellar bone structure with external cortical bone (E), diploic bone (D), and internal cortical bone (I) all discernable. Triangles denote the original bone margins. Scale bars, 250 μm. Reproduced from Lu et al. (2016) with permission from Copyright 2016 Wiley.

Lu et al. (2016) reported a graphene hydrogel (MGH) membrane fabricated by multiple, face-to-face stacked chemically converted graphene (CCG) sheets. This multilayer nanostructure was robust and flexible, with an average tensile modulus close to the order of magnitude of that of rat skull. Removal of water molecules between the CCG layers resulted in the irreversible collapse of the multilayer microstructure to form a more compact structure. In a typical rat cranial defect model, the authors verified that the MGH membrane could act as a barrier membrane for guiding bone regeneration. Micro-CT and histological analysis demonstrated the potential of the membrane to promote early osteogenesis and accelerate regenerative mineralization of mature lamellar bone (Figure 4). Lu et al. (2013) designed a self-supporting graphene hydrogel (SGH) film as an experimental platform to evaluate the biomedical properties of graphene particularly for bone regeneration. This strategy provided a valuable information for developing further applications of graphene in bone tissue engineering. Prakash et al. (2020) prepared a series of nanocomposite films containing GO, CS, HAP, polyvinyl alcohol (PVA), and gold for bone tissue engineering. The CS/PVA/GO/HAP/Au film showed good biocompatibility and osteogenic differentiation ability. The antimicrobial analysis demonstrated its significant inhibition against both Gram-positive and Gram-negative bacteria.

**FIGURE 5:**
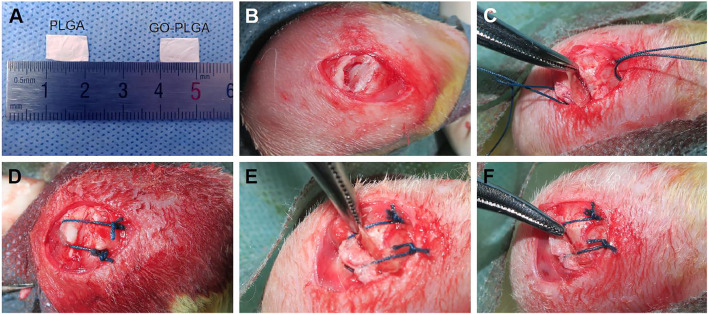
**(A)** General view of PLGAand PLGA-GO membranes and **(B–F)** surgical procedure of interposition of PLGAand PLGA-GO membranes in the rabbit supraspinatus tendon repair model. Reproduced from Su et al. (2019) with permission from Copyright 2019 Dove Medical Press Ltd.

By incorporating graphene nanoplates into poly (lactic-co-glycolic acid) (PLGA), Wu et al. (2018) fabricated a biofilm with osteogenic activity. The composite film was observed to enhance alkaline phosphatase (ALP) activity, calcium mineral deposition and osteogenesis-related gene expression levels. The activation of PI3K/Akt/GSK-3β/β-catenin signaling pathway by graphene may be the mechanism behind its osteoinductive properties. Pazarçeviren et al. (2019) fabricated a bilayer membrane through the covalent bonding of a dense polycaprolactone-polyethylene glycol-polycaprolactone (PCEC) membrane layer and a hydrogel layer, which was composed of bismuth doped bioactive glass (BG, 45S5) and graphene oxide (GO) particles incorporated in gelatin. The membrane could fill cavities and prevent soft tissue invasion, thus providing a barrier function for months. It also showed good osteoinductivity, osteoconductivity, high-resorbability and flexibility, thus creating a favorable microenvironment for bone regeneration. Su et al. (2019) prepared a highly interconnective nanofibrous membrane by electrospinning technique with GO and electrospun poly (lactide-co-glycolide acid) (PLGA). In a rabbit supraspinatus tendon repair model, membranes were implanted in the gap between the tendon and the bone. Compared with the PLGA group, GO-PLGA membrane could promote the tendon healing and bone regeneration, which significantly improved the collagen alignment and biomechanical properties (Figure 5).

### Implants Coated with GDs

GDs have been used as coatings for implants to improve durability and mechanical properties (Shin et al., 2017; Tobin, 2017; Madni et al., 2018). Many scaffolds including metals, inorganic nonmetals, natural or synthetic polymers can be coated with GDs to better adapt to the load-bearing environment of bone tissue (Lee et al., 2008). At the same time, surface properties of implants such as porosity, hydrophilicity, biomineralization ability, cell adsorption can be enhanced to improve the interaction between implants and bone tissue interface (De Marco et al., 2017). In addition, the osteoconductive and osteoinductive properties of GDs facilitate the new bone formation and promote the new bone integration with the surrounding bone tissue (Erezuma et al., 2021).

Inspired by the natural layer-by-layer assembly process, Guo et al. (2019) developed a multifunctional tissue scaffold with porous polyurethane as the matrix and a mixture of nanoscale CS and GO as the coating. CS and GO nanosheets were alternately held together by powerful electrostatic interactions, forming a robust multilayer structure to encase the polyurethane substrate. The authors were able to control the orientation and chemical composition of structural elements at the nanoscale and fill them with drug components. This multifunctional material could repair bone defects while allowing for drug release in response to pH changes, thus enabling potential multimodal therapeutic applications. Lee et al. (2015) explored the effect of rGO-coated HAP composites on osteogenic differentiation of BMSC. Using ALP activity and calcium-phosphate mineralization as early and late markers of osteogenic differentiation, respectively, this study confirmed that rGO synergistically enhanced the spontaneous osteogenic differentiation of human BMSC when wrapped around HAP particles. In addition, Zhao et al. (2015) explored the preparation, characterization, and cellular behavior of GO coatings on quartz substrates. These coatings with uniform thickness were prepared by a modified dip-coating procedure. Compared with the non-coated substrata and tissue culture plates, higher levels of ALP activity and osteocalcin secretion were revealed on the GO-coated substrates, while no significant differences in cytotoxicity, viability, proliferation and apoptosis were observed. Miyaji et al. (2014) wrapped the collagen scaffolds with GO and rGO, and then examined the bioactivity of GO and rGO films respectively. Compared with the non-coated group, GO- and rGO-coated groups showed significant increases in compressive strength and bioactivity. Moreover, rGO-coated scaffolds were more bioactive than GO-coated scaffolds due to their higher tissue ingrowth rate and better enhancement of calcium absorption and ALP activity.

**FIGURE 6:**
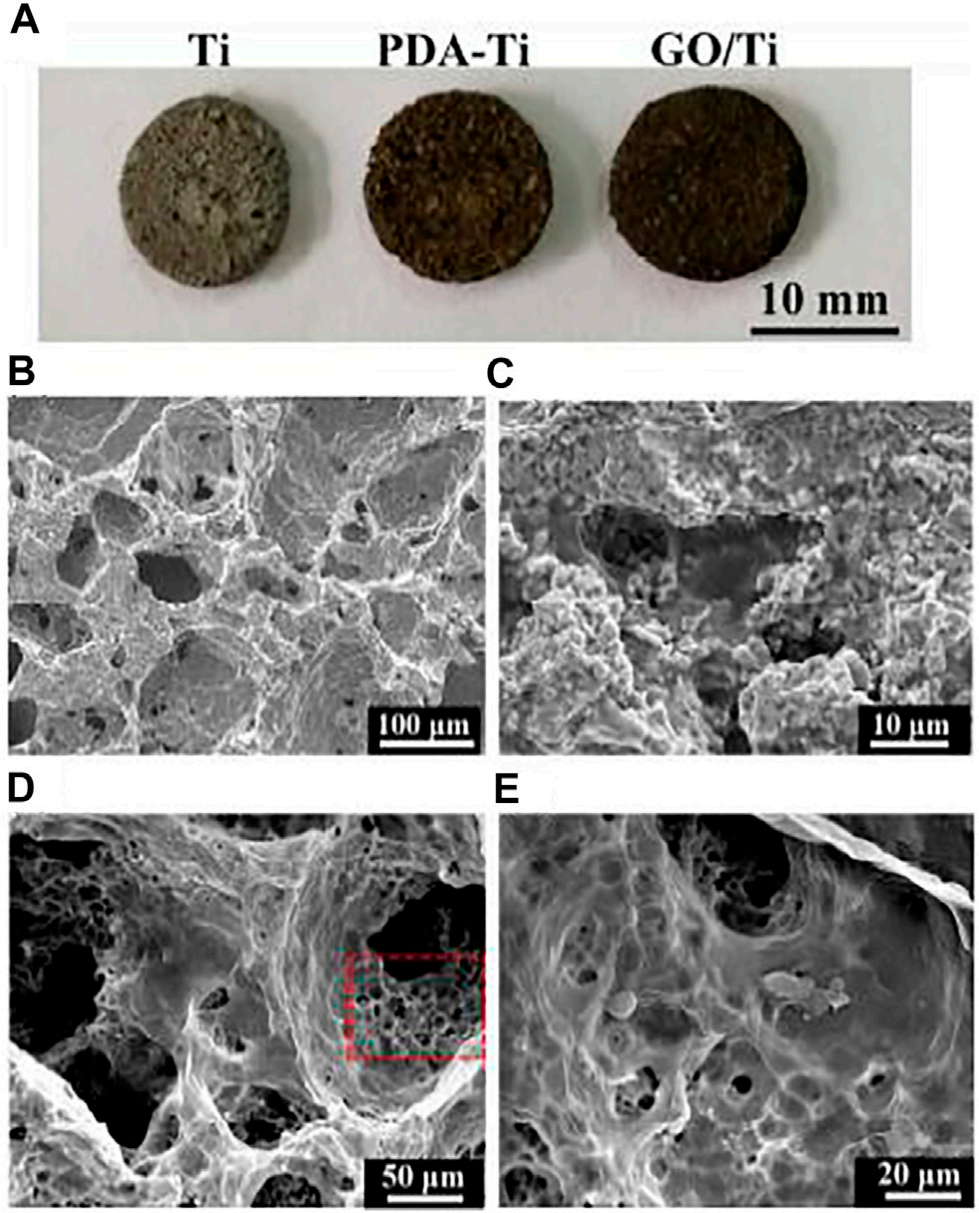
**(A)** Photo of the pure Ti, PDA modified Ti (PDA-Ti), and GO-wrapped Ti scaffolds. **(B)** SEM micrographs showing micropores in the Ti scaffold. **(C)** PDA ad-layer was coated on the surface of the Ti scaffold. **(D)** GO uniformly covered on the PDA-Ti scaffold. **(E)** Magnified image of **(D)**, showing wrinkled GO nanosheets wrapped in the pores of the Ti scaffold. Reproduced from Han et al. (2018b) with permission from Copyright 2018 Royal Society of Chemistry.

Titanium (Ti)-based endosseous implants have been widely used for a variety of bone defects and conditions because of their suitable mechanical properties, biocompatibility, and chemical stability (Rad et al., 2013; Abdel-Hady Gepreel and Niinomi, 2013; Qiang et al., 2021). However, weak osteoinductivity and osteoconductivity result in a lack of integration of the Ti scaffolds with the surrounding bone tissue (Li et al., 2015). This situation is expected to be improved by the addition of GDs-coating. To overcome the challenge of uniformly depositing GO on chemically inert Ti scaffolds, Han et al. (2018b) designed and developed a strategy by inspiration of mussels. Polydopamine (PDA) mediated the interaction between GO and Ti surfaces, thereby resulting in a homogeneous coverage of GO on Ti scaffolds (Figure 6). The nanostructure and functional groups of GO enabled the delivery of biomolecules and provided sites for cell adhesion, which provided a nanostructured environment for bone regeneration. Li et al. (2017b) further explored the effect of graphene coating on the bioactivity of Ti alloy (Ti6Al4V), which was widely used for hip and knee joint replacements. The final results showed that the cell proliferation rate and the level of osteoblast-specific gene transcription of graphene-coated Ti6Al4V were significantly increased.

**FIGURE 7:**
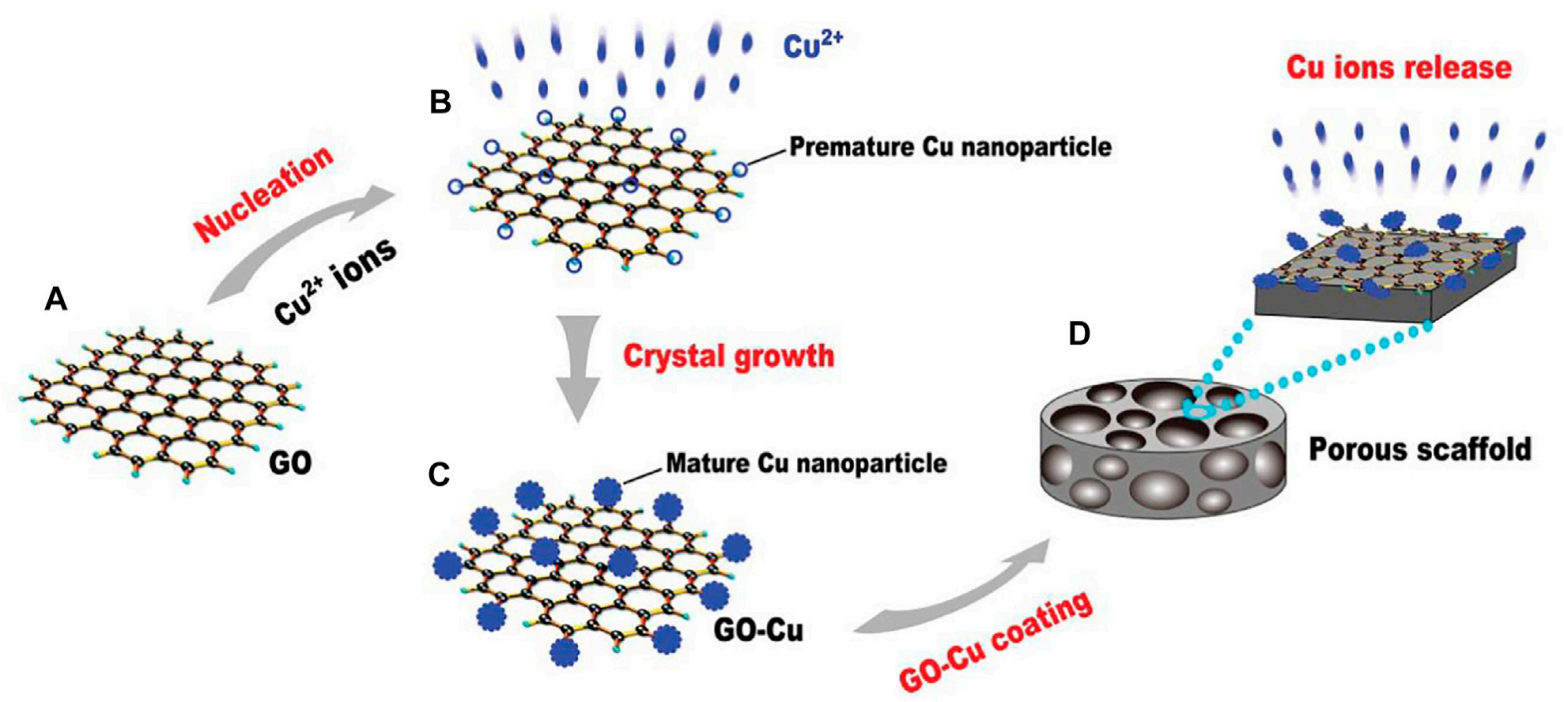
Schematic of the experimental protocol for the fabrication of GO-Cu-coated CPC scaffolds. **(A)** The pattern of GO. **(B)** Premature Cu nanoparticles on GO film. **(C)** Mature nanoparticles on GO film. **(D)** GO-Cu coated porous CPC scaffold. Reproduced from Zhang et al. (2016) with permission from Copyright 2016 Wiley.

Zhang et al. (2016) fabricated the water-soluble GO-copper nanocomposites (GO-Cu) as coating for porous calcium phosphate (CaP) scaffold (Figure 7). The composite material could be uniformly distributed on the scaffold surface and maintain the long-term release of copper ions. The GO-Cu-coated CaP scaffolds significantly promoted the angiogenesis and osteogenesis after implanting into the critical-sized rat cranial defects. Santos et al. (2015) prepared a multifunctional biodegradable coating material by hybridization of GO and HAP nanoparticles. The coating was then deposited on the ultra-high purity magnesium surface by a parallel nano assembling process. The surface properties of the coating can be tailored by adjusting the content of GO and HAP. Thereby, appropriate hydrophilicity, degradability, and surface mineralization could be obtained. The cobalt-chromium-molybdenum-based alloy (CoCrMo) was also an important candidate for orthopedic implants due to its excellent corrosion and wear resistance. Nonetheless, their biocompatibility and bioactivity were unsatisfactory (Lohberger et al., 2020). Although many attempts have been made to improve their biocompatibility, none of the efforts are effective (Poh et al., 2011; Logan et al., 2015; Sahasrabudhe et al., 2021). Zhang et al. (2018) intended to propose a solution to this challenge. Though an improved wet transfer approach, graphene was transferred to the surface of the alloy. Ultimately, *in vitro* experiments showed the improved biocompatibility and bioactivity of the graphene-coated CoCrMo alloy.

## Summary and Prospect

The ultimate goal of bone tissue engineering is to achieve restoration and reconstruction of human bone tissue to meet clinical treatment requirements, including but not limited to bone defects caused by trauma, infections, sarcomas and metabolic diseases (Holzapfel et al., 2013; Jakob et al., 2013; Fernandez-Yague et al., 2015). Bone regeneration is a complex and dynamic physiological process which macroscopically involves local mechanical stability, environmental matrix, and blood supply, and microscopically involves the interaction of multiple cells, signaling molecules, and effectors in a spatiotemporal sequence (Winkler et al., 2018). Therefore, an ideal bone graft substitute should exhibit the following characteristics: 1) good biocompatibility and non-immunogenicity to ensure safe clinical application; 2) suitable mechanical properties, including strength, viscoelasticity, toughness, and wear resistance to match the properties of host bone and ensure adequate longevity; 3) porous structure or rough surface to facilitate the ingrowth of cells and tissues; 4) favorable osteoconductivity and osteoinductivity to promote new bone production; 5) controlled degradability to match the rate of new bone ingrowth; 6) facile modifiability to meet the specific functional requirements of different application scenarios.

GD’s outstanding properties make it as one of the most anticipated materials for bone tissue engineering (Guo and Dong, 2011; Ding et al., 2015). Mechanical properties including strength, stiffness, and flexibility can best reflect the irreplaceability of GDs in bone repair. The mechanical reinforcement of GDs on the bone repair composites can be adjusted by the selection of different derivatives or by changing their content. GDs-based composites possess favorable viscoelasticity that can be molded as needed to better adapt to the physical characteristics of bone tissue. GDs also has strong structural stability and is less likely to be destroyed during complex preparations as well as in the physiological environment of the implant site. In addition, good electrical conductivity of GDs can not only directly stimulate cellular osteogenic activity, but also indirectly adsorb active factors to promote bone formation (Turk and Deliormanlı, 2017; Dalgic et al., 2018). Conductivity can also be used for signal control or magneto-thermal therapy in special cases, such as focal clearance for osteosarcoma or local infections. Moreover, GDs can promote biomineralization and bone-like apatite formation on the implant surface, thus enhancing osseointegration and osteoconductivity. Planar structure and large specific surface area impart GDs with excellent ability to immobilize various biomolecules, cells, drugs and other desired substances (Wang et al., 2011; Shen et al., 2012; Feng et al., 2013; Yang et al., 2013). On the other hand, such structure makes it easier for GDs to be modified with multifunctional groups, which in turn significantly improve the dispersibility and hydrophilicity of GDs in the composites. Based on the above advantages, GDs have become an indispensable component of bone repair composites in many studies.

Although promising progress has been made in current research of GDs-based materials, there are still many challenges to be faced before clinical application. Firstly, taking into account the cost-effectiveness and accessibility, the production and processing techniques of GDs are yet to be broken through. At present, the broad application of GDs is restricted due to the difficulty of large-scale synthesis. Cost may become a constraint for further research. Furthermore, the problem of GD’s aggregation in solution during the fabrication of composites also remains to be solved (Xu et al., 2016; Syama et al., 2017). How to promote the homogeneous distribution of GDs in the matrix will also be a focus of future research. Secondly, the long-term safety of GDs-based materials for *in-vivo* use is still unclear. It is imperative to gain insight into the interactions of GDs with biological systems. There have been concerns regarding its biocompatibility and toxicity, but convincing, high-quality studies are still insufficient. The observation period of existing studies is not long enough, so chronic toxicity studies longer than 6 months should be encouraged; on the other hand, the most of adopted animal models were rats and rabbits, lacking large animal models such as pigs, goats and monkeys. What’s more, the mechanism of interaction between GDs and the *in vivo* environment after implantation needs to be further clarified. Exploration of osteogenic mechanisms may point out the right direction for further study; analysis of cellular uptake and response mechanisms facilitates the prediction of acute and chronic adverse reactions; then the clarification of degradation and metabolism mechanisms will help to understand the spatiotemporal distribution of GDs *in vivo*. In summary, in spite of various challenges, GDs are likely to be a real breakthrough for future research in regenerative medicine, and advances in related technologies will pave the way for earlier clinical use of GDs.
